# Schooling of the patients and clinical application of questionnaires in osteoarthitis

**DOI:** 10.1590/1413-78522014220500980

**Published:** 2014

**Authors:** Gustavo Constantino De Campos, Marcelo Tomio Kohara, Marcia Uchoa Rezende, Olga Fugiko Magashima Santana, Merilu Marins Moreira, Olavo Pires De Camargo

**Affiliations:** 1 Universidade de São Paulo, Faculdade de Medicina, Hospital das Clínicas, São Paulo, SP, Brazil, Instituto de Ortopedia e Traumatologia, Hospital das Clínicas da Faculdade de Medicina da Universidade de São Paulo, São Paulo, SP, Brazil

**Keywords:** Osteoarthritis, Questionnaires, Knee joint

## Abstract

**Objective:**

To evaluate the consistency of the questionnaires (WOMAC, Lequesne, VAS, SF 36-PCS and SF 36-MCS) when applied in patients with osteoarthritis of the knees (KOA) verifying if age and level of education interfere with the completion of the questionnaires.

**Method:**

One hundred and two patients with KOA answered WOMAC, LESQUESNE, VAS and SF-36 questionnaires and provided data correlated with age and education. The internal consistency of the WOMAC questionnaire was verified with Cronbach's alpha. Pearson's correlations between the questionnaires, age and educational level was performed.

**Results:**

Mean age was 65 years old. Schooling averaged 7.94 years; WOMAC 47.95; VAS 63.57; Lequesne 12.29; PCS and MCS 31.91 43.68. Cronbach's alpha for WOMAC 0.9. Education did not affect WOMAC response (r=-0.182, p = 0.067) and MCS (r=0.021 / p=0.835), but showed weak but significant correlation with VAS (r=-0.264 / p=0.007), Lequesne (r=0.277, p=0.005) and PCS (r=0.309/ p=0.002). Age showed significant direct correlation only with PCS (r=0.205, p=0.039).

**Conclusion:**

The level of education does not interfere with the completion of WOMAC but may interfere with completing VAS, Lequesne and physical component of SF-36. *Level of Evidence II, Retrospective Study.*

## INTRODUCTION

In research and in clinical practice it is essential, for the diagnosis of diseases and to assess changes in the patients' condition over time, some form of measurement. Measurement is the foundation of most clinical decisions in medicine.^1^ There is currently a plethora of questionnaires and evaluation measurements used in clinical practice in patients with various musculoskeletal diseases.^2^ However, there is not yet a well-defined package of assessment tools to determine whether the treatment is being effective symptomatically and economically, or even one that facilitates the communication between various health agents.^2^

Osteoarthritis, an extremely prevalent disease,^3^^,^ 4 is now considered a burden to society, due to individual, social and economic impact.^5^ It is a problem of great complexity and difficult to understand and manage, because it deals not only with painful symptoms, but also with physical and psychosocial changes, causing disability, difficulty in daily activities, loss of independence and poor quality of life,^6^ besides a huge financial loss to society.^7^ The evaluation of the degree of injury and outcome of patients with osteoarthritis is currently done through radiographic classifications, pain scales, questionnaires assessing physical, functional, psychological and general aspects, such as quality of life.^8^^,^ 9

Agreement regarding the use of outcomes in clinical trials is extremely important to facilitate comparisons and provide appropriate estimates of benefit and safety of therapeutic interventions in different patient populations. "Clinical studies are as reliable as their outcomes".^10^ In this context, the Brazilian population represents a huge challenge regarding measurement capability of various questionnaires, given the cultural, social and educational heterogeneity of our country. Although we rely on instruments validated for the Portuguese language,^11^^-^ 13 its consistency and reliability are put in check when the population presents low socio-educational level, a common characteristic of patients attending Brazilian university hospitals. This study, therefore, aims to assess whether the consistency of the questionnaires used in patients with osteoarthritis (WOMAC,

Lequesne, VAS and SF-36) is influenced by age and educational level of patients.

## METHODS

This study was conducted at the Department of Orthopedics and Traumatology, *Instituto de Ortopedia e Traumatologia*,* Hospital das Clínicas da Faculdade de Medicina da Universidade de São Paulo*, (DOT-IOT-HCFMUSP). It has been approved by the Ethics Committee for Analysis of Research Projects (CAPPesq) under N° 0622/11.

The records of 102 individuals, patients who were being treated for osteoarthritis in the knees through the PARQVE (Osteoarthritis Project Recovering Quality of Life through Education) program were analysed.^14^ Medical records of patients with osteoarthritis according to clinical and radiologic criteria^15^ which contained information of interest for this project were included in the study. The information evaluated were age, education (years of schooling), visual analog pain scale (VAS), WOMAC, Lequesne and SF-36 questionnaires, the later including physical (PCS) and mental (MCS) components.

The evaluated scales, age and education were described by using means and standard deviation with the confidence intervals of 95% for the means.^16^ The internal consistency of the WOMAC questionnaire using Cronbach's alpha and the correlations of each item comprising the questionnaire was verified with the total scale when the item is removed. The Cronbach's alpha of the total questionnaire was also calculated. Pearson correlations^16^ were calculated between the assessed scales and also with the age and education of patients to verify if the scales correlate with each other and whether age and education influence the results of the scales. Scatter plots were constructed by adjusting the estimated line in order to evaluate the influence of education on the outcome of the questionnaires. The tests were performed with a significance level of 5%.

## RESULTS

Table 1 shows that the mean age of patients was 65 years old (St.Dev.=10.8 years old), the scales of quality of life showed average values below 50% and the rating scales of osteoarthritis showed average values near half of the values of the scales, indicating a somehow-functional impairment of these patients.

**Table 1:** Description of age, level of education and scales evaluated by patients.

**Variable**	**Mean**	**St. Dev**	**CI (95%)**
Age (years old)	65.13	10.79	63.04 - 67.22
Education (years)	7.94	3.04	7.35 - 8.53
WOMAC Pain	9.29	3.79	8.55 - 10.03
WOMAC Stiffness	3.78	2.1	3.37 - 4.19
WOMAC Function	34.87	12.77	32.39 - 37.35
WOMAC Total	47.95	17.16	44.62 - 51.28
VAS	63.57	26.43	58.44 - 68.70
Lequesne	12.29	4.24	11.47 - 13.11
PCS	31.31	8.26	30.31 - 33.52
MCS	43.68	12.35	41.28 - 46.08

Table 2 shows that the items of the WOMAC questionnaire correlated between 0.6 and 0.8 with the total questionnaire when removed from it, indicating that they contribute significantly to the assembly of the questionnaire in this population, without influencing the final outcome of the questionnaire. Cronbach's alpha values were all higher than 0.9 with the total of the items of the questionnaire; when the item was removed from the questionnaire and also the total WOMAC showed a Cronbach's alpha of 0.943, showing that the items which integrate the questionnaire, when applied to this population, have largely contributed to the questionnaire as a whole and similarly.

**Table 2:** Result of assessment of internal validity of WOMAC questionnaire applied to patients.

**Item**	**Mean of** **scale if item** **was removed**	**St. Dev of** **scale if item was removed**	**Correlation of** **item with the** **remaining scale**	**Cronbach's** **alpha if item** **was removed**
WD1	46.29	16.44	0.664	0.940
WD2	45.52	16.67	0.476	0.943
WD3	46.62	16.46	0.582	0.941
WD4	46.90	16.60	0.560	0.942
WD5	45.98	16.48	0.662	0.940
WR1	46.06	16.49	0.551	0.942
WR2	46.42	16.39	0.619	0.941
WF1	45.70	16.50	0.621	0.941
WF2	45.59	16.62	0.555	0.942
WF3	45.91	16.42	0.625	0.941
WF4	46.03	16.48	0.621	0.941
WF5	45.41	16.62	0.473	0.943
WF6	46.48	16.50	0.612	0.941
WF7	45.79	16.52	0.575	0.942
WF8	46.07	16.40	0.634	0.941
WF9	46.09	16.32	0.658	0.940
WF10	46.05	16.31	0.739	0.939
WF11	46.11	16.34	0.684	0.940
WF12	46.80	16.47	0.654	0.941
WF13	46.76	16.34	0.709	0.940
WF14	46.66	16.38	0.689	0.940
WF15	45.93	16.30	0.740	0.939
WF16	45.22	16.63	0.526	0.942
WF17	46.34	16.51	0.673	0.940
WOMAC Total				0.943

**Figure 1:**
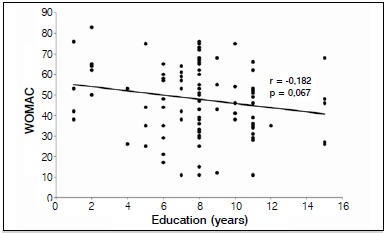
Scatter diagram between education and total WOMAC.

**Figure 2:**
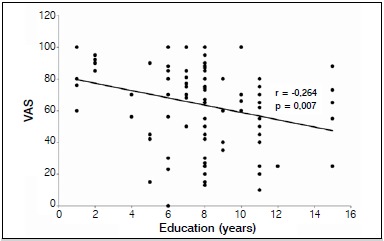
Scatter diagram between education and VAS.

**Figure 3:**
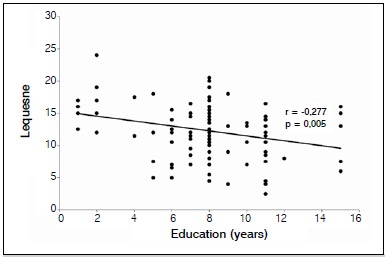
Scatter diagram between education and Lequesne.

**Figure 4:**
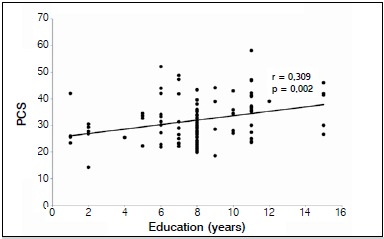
Scatter diagram between education and PCS.

Figure 1 suggests randomness of WOMAC regarding the education of patients, the correlation obtained was -0.182

(p=0.067). Figures 2-4 show little relationship of VAS, Lequesne and PCS scales with the education of patients, however, it is observed that, although significant (p<0.05), the correlations presented were close to zero.

**Figure 5:**
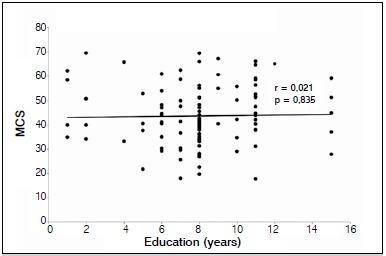
Scatter diagram between education and MCS.

Figure 5 shows lack of influence of age on mental component of quality of life r = 0.021 (p = 0.835).

We see in Figures 2-5 that, as already mentioned, education correlated significantly with VAS, Lequesne and PCS.

The scales evaluated showed significant correlations with all other scales that assess function coherently, i.e., direct

correlations between WOMAC, VAS and Lequesne scales. (Table 3) Only PCS showed inverse correlations with the other scales, since the larger the score of that scale, the better the patient shows, while in the other scales this relationship is reversed.

**Table 3:** Result of correlations between scales, age and education.

**Correlation**		Age(years old)	Education(years)	Total pain	Total stiffness	Total function	WOMAC Total	VAS	Lequesne	PCS
Education(years)	r	-0.142								
	p	0.155								
WOMAC pain	r	-0.135	-0.140							
	p	0.176	0.161							
WOMAC stiffness	r	-0.089	-0.143	0.602						
	p	0.376	0.151	<0.001						
WOMAC function	r	0.017	-0.179	0.736	0.595					
	p	0.867	0.071	<0.001	<0.001					
WOMAC total	r	-0.028	-0.182	0.842	0.698	0.979				
	p	0.779	0.067	<0.001	<0.001	<0.001				
VAS	r	-0.117	-0.264	0.604	0.402	0.489	0.547			
	p	0.24	0.007	<0.001	<0.001	<0.001	<0.001			
Lequesne	r	0.153	-0.277	0.563	0.507	0.621	0.648	0.496		
	p	0.125	0.005	<0.001	<0.001	<0.001	<0.001	<0.001		
PCS	r	0.205	0.309	-0.512	0.634	-0.496	-0.527	-0.463	-0.525	
	p	0.039	0.002	<0.001	<0.001	<0.001	<0.001	<0.001	<0.001	
MSC	r	-0.068	0.021	-0.182	-0.178	-0.165	-0.185	-0.151	-0.241	-0.065
	p	0.498	0.835	0.068	0.073	0.097	0.063	0.129	0.015	0.518


Result of Pearson correlation.

## DISCUSSION

It is of significant importance the proper perception and evaluation of patients with knee osteoarthritis, both for analysis of quality of life, and for determining therapeutic measures.^17^^,^ 18 Today, most of the instruments that were used in the English language have been translated into Portuguese and adapted to Brazilian conditions.^19^ However, we know that besides careful translation, each physical and mental measurement must be contextualized into the specific culture of its society, respecting its attitudes, beliefs, behaviors and social habits.

In our experience treating Brazilian patients, many of them lacking adequate literacy and proper basic education, we had the impression that the age and lack of schooling compromised the understanding of the research instruments and consequently, their answers and filling out. With this question in mind, our study aimed to find out if questionnaires applied to the population are consistent and verify the influence of age and education on the results of various research instruments.

The mean age was 65 years old (St. Dev.=10.8 years old) and the average values of the questionnaires results showed expected values for chronic illnesses, with an average value indicating compromised pain and quality of life of patients. The high value of matching components of WOMAC (0.6 to 0.8) and Cronbach's alpha (>0.9) show that the instrument items are all and similarly important.

The number of patients analyzed was sufficient for a statistically significant sample (p<0.05). However, the scatter diagram of instruments *versus* education showed low correlations

(r =-0.182 for WOMAC, r=-0 .264 for VAS, r=-0.277 for Lesquesne, r=0.309 for PCS and r=0.021 for MCS). In the results, age showed statistically significant direct correlation only with PCS (r=0.205 and p=0.039), but also the value of the correlation was very close to zero. This means that all questionnaires were suitable to evaluate patients with knee osteoarthritis,

irrespective of age or education.

The WOMAC questionnaire is regarded as one of the most used and suitable for the assessment of the limitations related to physical aspects of knee OA.^20^^,^ 21 Our results indicate that schooling did not interfere with its use and, hence, can be used for evaluation this group of patients. Visual analog scale questionnaires for pain (VAS) should be used with caution, because pain scales unrelated to the functional performance denotes an isolated and risky factor, since patients who feel pain to perform a function have a tendency to reduce that function for symptomatic relief.

Although the results show that we can rely on questionnaires despite level of education and age, we noticed a visible difference in the understanding of the research process. Patients who lack basic school education required explanations repeatedly and required a greater time of consultation and final evaluation, which required larger effort by the team during interviews and the fill out of questionnaires. In many cases, particularly in the application of SF-36 (PCS/MCS), the difficulty in understanding each item lead us to use explanatory and similar examples, that could even slightly modify the patients' responses. 

Our impression is that all questionnaires are dependent on a full understanding by the patient and the investigator. But variables such as understanding pain and function were dependent on a certain level of reasoning organization to be properly measured. This was very visible in the difficulty of implementing visual analog pain scale (VAS) by patients with lower level of education, requiring repetition of the questionnaires, new explanations and exemplification, those not exempt from the risk of bias due to failures to understanding. There are patients with low educational level that present logical reasoning and can, if explained, transcribe their pain on a 100 mm ruler, marking numbers on the intensity of pain. But there are others with serious difficulties in explaining pain in an analog form. The low correlation between VAS, Lequesne and SF-36 with schooling can be explained by being not only a reflection of education, but of intelligence quotient, or logical reasoning. This question remains to be answered by further study.

## CONCLUSION

Finally, we believe that the tools can be used among the Brazilian population with knee osteoarthritis, regardless of educational level and age, however, with exceptions. Every patient should have an individualized consultation and evaluation, especially in cases of analog pain scale (VAS). Patients with low educational level require more detailed explanation and support with an easier and more simplistic language, sometimes with more examples.

## References

[1] Lassere MN (2006). A users guide to measurement in medicine. Osteoarthritis Cartilage.

[2] Howe TE, Dawson LJ, Syme G, Duncan L, Reid J (2012). Evaluation of outcome measures for use in clinical practice for adults with musculoskeletal conditions of the knee: a systematic review. Man Ther.

[3] Lawrence RC, Felson DT, Helmick CG, Arnold LM, Choi H, Deyo RA (2008). Estimates of the prevalence of arthritis and other rheumatic conditions in the United States. Part II. Arthritis Rheum.

[4] Dillon CF, Rasch EK, Gu Q, Hirsch R (2006). Prevalence of knee osteoarthritis in the United States: arthritis data from the Third National Health and Nutrition Examination Survey 1991-94. J Rheumatol.

[5] Bitton R (2009). The economic burden of osteoarthritis. Am J Manag Care.

[6] Rezende MU, Campos GC, Pailo AF (2013). Current concepts in osteoarthritis. Acta Ortop Bras.

[7] Le TK, Montejano LB, Cao Z, Zhao Y, Ang D (2012). Health care costs in US patients with and without a diagnosis of osteoarthritis. J Pain Res.

[8] Tsai PF, Tak S (2003). Disease-specific pain measures for osteoarthritis of the knee or hip. Geriatr Nurs.

[9] Brooks P, Hochberg M (2001). Outcome measures and classification criteria for the rheumatic diseases. A compilation of data from OMERACT (Outcome Measures for Arthritis Clinical Trials), ILAR (International League of Associations for Rheumatology), regional leagues and other groups. Rheumatology (Oxford).

[10] Tugwell P, Boers M (1993). OMERACT conference on outcome measures in rheumatoid arthritis clinical trials: introduction. J Rheumatol.

[11] Fernandes MI (2003). Tradução e validação do questionário de qualidade de vida específico para osteoartrose WOMAC (Western Ontario McMaster Universities) para a língua portuguesa.

[12] Marx FC, Oliveira LM, Bellini CG, Ribeiro MCC (2006). Tradução e validação cultural do questionário algofuncional de Lequesne para osteoartrite de joelhos e quadris para a língua portuguesa. Rev Bras Reumatol.

[13] Ciconelli RM (1997). Translation and validation of the Portuguese of the Medical Outcomes Study 36-Item Short-Form Health Survey (SF-36).

[14] Rezende MU, de Campos GC, Pailo AF, Frucchi R, Pasqualin T, Camargo OP (2013). PARQVE-Project Arthritis Recovering Quality of Life by Means of Education Short-term Outcome in a Randomized Clinical Trial. J Arthritis.

[15] Altman R, Asch E, Bloch D, Bole G, Borenstein D, Brandt K (1986). Development of criteria for the classification and reporting of osteoarthritis. Classification of osteoarthritis of the knee. Diagnostic and Therapeutic Criteria Committee of the American Rheumatism Association. Arthritis Rheum.

[16] Kirkwood BR, Sterne JAC (2006). Essential medical statistics.

[17] Faden R, Leplége A (1992). Assessing quality of life. Moral implications for clinical practice. Med Care.

[18] Guyatt GH, Feeney DH, Patrick DL (1993). Measuring health- related quality of life. Ann Intern Med.

[19] Guillamin F (1995). Cross-cultural adaptation and validation of health status measures. Scand J Rheumatol.

[20] Roos EM, Roos HP, Lohmander LS (1999). WOMAC osteoarthritis index - additional dimensions for use in subjects with post-traumatic osteoarthritis of the knee Western Ontario and MacMaster Universities. Osteoarthritis Cartilage.

[21] Roos EM, Toksvig-Larsen S (2003). Knee injury and osteoarthritis outcome score (KOOS) - validation and comparison to the WOMAC in total knee replacement. Health Qual Life Outcomes.

